# On the normative roles of biodiversity and naturalness in conservation

**DOI:** 10.1111/cobi.70072

**Published:** 2025-05-31

**Authors:** David Saltz, Shlomo Cohen

**Affiliations:** ^1^ Mitrani Department of Desert Ecology, Swiss Institute for Dryland Environment and Energy Research Ben Gurion University of the Negev Midreshet Ben‐Gurion Israel; ^2^ Department of Philosophy Ben‐Gurion University of the Negev Beer Sheva Israel

**Keywords:** biodiversity, ecocentric, ethics, homeostasis, intrinsic value, naturalness, nature, pluralism, principle pluralism, ecocéntrico, ética, homeostasis, pluralismo, principios, natural, naturaleza, valor intrínseco, **关键词**: 迁徙屏障, 陆鸟迁徙, 土地利用变化, 雷达鸟类学, 停歇与通过比率(SPR)、风向选择, 墨西哥湾, 玉米带

## Abstract

*Nature* is an opaque concept. Consequently, the term *biodiversity conservation* has replaced *nature conservation* in most conservation contexts. We review the conceptual indeterminacies that plague the terms *nature* and *natural* but then show that comparable difficulties plague *biodiversity*. Then, we provide a new theory that sorts out the respective normative roles of naturalness and biodiversity within the ecocentric–intrinsic school of conservation. This is an elaboration on the conservation philosophy presented by Saltz and Cohen (2023). They presented a 3‐tiered normative scheme: ultimate value, midlevel principles, and lower level case‐specific judgments. The ultimate value is naturalness, which exists on a gradient. Ethical judgment is needed to choose the most adequate midlevel principle or principles among autonomy, integrity, and resilience based on case‐specific parameters and the goal of maximizing naturalness in a given area. Saltz and Cohen (2023) do not specify the role of biodiversity, however. We fill in that crucial gap by explaining that the midlevel principles refer to structural and functional biodiversity. The principles prioritized are those that will contribute the most to naturalness, depending on the biodiversity attributes and management options in a given area. In this scheme, biodiversity represents the lower tier, case‐specific metrics for assessing naturalness. However, because biodiversity can only be quantified by proxies that cannot be projected onto a unified scale, *biodiversity* acts as an umbrella term for the measures that are the metrics for assessing naturalness. As such, biodiversity is a salient parameter to be measured for maximizing naturalness in conservation and is analogous to measures of homeostasis for safeguarding health in medicine.

## Abstract (es)

Los papeles normativos de la biodiversidad y la naturalidad en la conservación

**Resumen:**

El concepto de *naturaleza* no es muy claro. Por esto, el término *conservación de la biodiversidad* ha sustituido al de *conservación de la naturaleza* en la mayoría de los contextos de conservación. Revisamos las indeterminaciones conceptuales que plagan los términos *naturaleza* y *natural*, pero a continuación mostramos que las dificultades comparables plagan la *biodiversidad*. En consecuencia, presentamos una nueva teoría que ordena los respectivos papeles normativos de la naturalidad y la biodiversidad dentro de la escuela ecocéntrica‐intrínseca de la conservación. Esta es una elaboración de la filosofía de la conservación presentada por Saltz y Cohen (2023), quienes presentaron un esquema normativo de tres niveles: valor último, principios de nivel medio y juicios específicos de casos de nivel inferior. El valor último es la naturalidad, que existe en un gradiente. El juicio ético es necesario para elegir el principio o principios de nivel medio más adecuados entre autonomía, integridad o resiliencia, basándose en parámetros específicos de cada caso y en el objetivo de maximizar la naturalidad en un área determinada. Sin embargo, Saltz y Cohen (2023) no especifican el papel de la biodiversidad. Nosotros cerramos ese vacío crucial explicando que los principios de nivel medio se refieren a la biodiversidad estructural y funcional. Los principios prioritarios son los que más contribuirán a la naturalidad, en función de los atributos de biodiversidad y las opciones de gestión de una zona determinada. En este esquema, la biodiversidad representa el nivel inferior, la métrica específica de cada caso para evaluar la naturalidad. Sin embargo, dado que la biodiversidad sólo puede cuantificarse mediante indicadores indirectos que no pueden proyectarse a una escala unificada, la *biodiversidad* actúa como término paraguas de las medidas que constituyen los parámetros para evaluar la naturalidad. Como tal, la biodiversidad es un parámetro destacado que debe medirse para maximizar la naturalidad en la conservación y es un concepto similar a las medidas de homeostasis para salvaguardar la salud en medicina.

## 评估玉米带对美国陆地鸟类迁徙造成的人为屏障

**【摘要】** 迁徙陆地鸟类会通过调整其飞行和停留行为, 以有效地穿越如墨西哥湾和撒哈拉沙漠等不宜居留的地域。除了这些自然障碍外, 鸟类还可能会越来越多地遇到因大规模土地利用变化而造成的人为障碍。其中一个例子是美国中西部的玉米带, 该地区76.4%的殖民前植被(包括森林和草地)已被农业和城市用地所取代, 主要为玉米地。本研究利用来自美国47个气象雷达站5年的数据, 比较了春秋两季穿越玉米带及其以南和以北森林景观的迁徙陆鸟种群水平的飞行模式。我们还通过比较迁徙鸟类停留休息的比例(停留与通过比率[stopover‐to‐passage ratio, SPR])相对于距离玉米带和墨西哥湾这两个屏障的变化, 分析了玉米带相较墨西哥湾对迁徙鸟类停留行为的影响。我们发现, 与森林景观相比, 鸟类在穿越玉米带时表现出更快的经向飞行速度和对顺风风向更强的选择。在迁徙个体穿越墨西哥湾的过程中, 随着与海岸线距离的增加, SPR急剧下降。在穿越墨西哥湾后, 停下来休息的迁徙个体所占比例最高, 且随着与海岸线距离的增加, SPR急剧下降。我们没有在穿越玉米带后的迁徙鸟类中发现类似的模式, 尽管在两个季节中, 当鸟类接近下行路线森林边界时, 在玉米带中的SPR都在增加。与海湾相比, 鸟类穿越玉米带后停歇倾向的模式较弱, 这可能是因为玉米带宽度较窄、在整个玉米带中存在小片森林, 以及受玉米带影响的物种较少。我们建议在玉米带中恢复森林作为垫脚石, 并保护墨西哥湾沿岸地区的林地, 以帮助陆地鸟类成功通过这两个屏障。**【翻译:胡怡思;审校:聂永刚】**

## INTRODUCTION

Of the 3 schools of moral thinking in natural‐science‐based conservation (ecocentric–intrinsic value, anthropocentric–instrumental value, and anthropocentric–relational value) (Pascual et al., 2021), the ecocentric–intrinsic school predominates among proenvironment actors (Taylor, Chapron, et al., 2020). Nevertheless, many normative and norm‐related key concepts in the ecocentric–intrinsic value school are ill‐defined and subject to ongoing debates (Callicott et al., 1999; Heath, 2021). Noteworthy is the debate regarding 3 key concepts: *nature*, *natural*, and *biodiversity*. Such debates hamper decision‐making in the realm and lobbying efforts in the political arena (Meinard et al., 2019).

**FIGURE 1:**
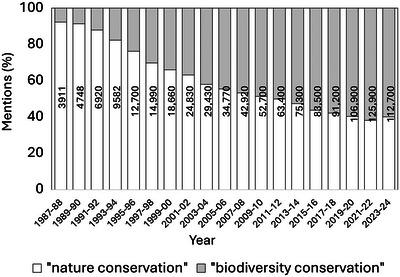
Change in the relative number of mentions of the exact phrases *nature conservation* and *biodiversity conservation* (the percentage of each term is relative to the sum of mentions for both) in papers published from 1987 to 2024 returned in a search for these exact phrases in Google Scholar (bars, 2‐year blocks; numbers in bars, total number of detected papers for the specified time frame).

Although a common term, the meaning of *nature* (and its adjective *natural*) in the framework of mainstream conservation science has been subject to continued controversy (e.g., the nature–culture divide [Uggla, 2010]), casting doubt on its usefulness (Deplazes‐Zemp, 2022; Plumwood, 2005; Williams, 1980). Consequently—especially since the first National Forum on BioDiversity in 1986—the term *biodiversity* has become a foundational conservation concept (Sarkar, 2002), progressively replacing *nature* in the context of conservation (i.e., biodiversity conservation instead of nature conservation) (Figure 1). This transition is mostly deemed beneficial to conservation efforts because *biodiversity* is argued by its proponents to be less ambiguous than *nature* and because it is easier to convey to the public in terms of its ecocentric and anthropocentric value and is, therefore, a more effective fighting tool in the political arena. Accordingly, biodiversity has become a rallying point around which conservation efforts are focused (Díaz & Malhi, 2022). Nevertheless, this transition is not without criticism (Santana, 2016). Specifically, it may be problematic by concealing a possible shift in goals. As Keune et al. (2022, p. 36) argue, “…referring to biodiversity instead of Mother Nature (or vice versa) imply different future worlds. This makes biodiversity governance a contested field….”

We address the conceptual and normative relations between *nature*, *natural*, and *biodiversity* within the framework of ecocentric–intrinsic value conservation thinking. The intrinsic versus instrumental debate and the question of the merit of ethical pluralism (Cortes‐Capano et al., 2022; Vucetich et al., 2015) are beyond the scope of this work. In the first part of the article, we review the difficulties that beset the concepts of *nature* and *natural* and argue that equivalent difficulties are associated with *biodiversity*, making the concept no less contentious. In the second part, we offer new ways to look at relations between *nature*, *natural*, and *biodiversity* in conservation philosophy based on the 3‐tiered moral framework described by Saltz and Cohen (2023).

## TENSIONS SURROUNDING *NATURE, NATURAL*, AND *BIODIVERSITY*

### *Nature* and *natural*

The meaning and perception of nature have evolved over time and culture (Lanzerath, 2014). In Western culture, for example (Lee, 2005), the Greeks conceived nature as an organism, followed by the post‐renaissance era when the idea emerged that nature could be considered as a machine, and finally, with the acceptance of evolution in the 19th century, nature was recognized as a temporal process of which humans are part. The contemporary meaning of *nature* is similarly ambiguous. Authors provide lists of multiple definitions, expressing a puzzle as to what nature is.

The key problem concerning *nature* in conservation is the nature–culture divide (i.e., whether humans are, or are not, a part of nature) (Plumwood, 2001; Uggla, 2010). This is the contrast between *nature* as “the whole of material reality that is independent of human activity and history” and “the whole universe […] of material phenomena…” (Ducarme & Couvet, 2020). This contrast may be an outcome of the shift from the historical view of nature as ‘the other’ (either organism or machine) to its evolutionary view. From a different perspective, some scholars view nature as an “omnipresent entity” (Hillebrecht & Berros, 2017), whereas others claim that nature has been destroyed by humans and no longer exists (McKibben, 1989). Yet, others argue that nature is a social construct that did not exist before the term was coined (see Everden [1992] vs. Rolston [1997]). The debate also revolves around what humanity's relationship with nature should be (Curry, 2008). If nature is autonomous (Lee, 2005), then it is sensible to advocate respecting its autonomy. If, conversely, nature is a human construct, then arguments for protecting nature may well be invalid (excepting anthropogenic nostalgia [Williams, 1980]). These conflicting views threaten to render the term *nature conservation*, defined as minimizing anthropogenic impact, nebulous (Williams, 1980), casting doubt on its usefulness and generating the perception that the term causes more harm than good (Newman et al., 2017; Vogel, 2015). Takacs (1996, p. 75) concludes that “…nature is so polymorphous a term that what one attributes to it may say more about the speaker than it does about the natural world.” Because the perception of nature varies among individuals and cultures (Sandbrook et al., 2019), many advocate a pluralistic attitude toward the ethical views of conserving nature (Keune et al., 2022; Lele, 2021; Tallis & Lubchenco, 2014) in the 3 schools of thinking (Pascual et al., 2017): ecocentric–intrinsic, anthropocentric–instrumental, and anthropocentric–relational. The ultimate value of the ecocentric school, with which we are concerned, is naturalness.

*Natural*, as an adjectival form of *nature*, is also subject to debate (Vogel, 2015). Its common definition is: free from anthropogenic, technology‐related (*technetos*, Greek = artificial) influence. *Artificial* is its counterconcept (Birnbacher, 2019; Hunter, 1996). The definition of *natural* is relatively unambiguous and enables valuation on a naturalness gradient (Angermeier, 2000). The extreme of complete naturalness is no longer attainable due to global anthropogenic impacts; thus, preservation (as opposed to conservation) is no longer feasible. Naturalness in this sense is often perceived as synonymous with nature's autonomy (Hettinger, 2005) and is commonly recognized as having inherent value (Nuffield Council on Bioethics, 2015; Tanner, 2009). However, the ambivalence surrounding its source noun *nature* extends to the definition of *natural* as well and generates a conundrum: if artificial is the counter concept of natural, then art (i.e., being the product and impact of human actions) must be the counter concept of nature. Yet, art is considered part of nature when understood as the totality of empirical phenomena (Mill, 1874). The meaning of *natural* also becomes contentious when considering the restoration of damaged ecosystems. Does the restoration itself, which constitutes further anthropogenic influence, reduce the naturalness of ecosystems or does it enhance the naturalness by benchmarking the ecosystem against some past less‐disturbed state (Siipi, 2008)?

### Biodiversity

The dissentions concerning *nature* and *natural* fostered a transition to *biodiversity* as a substitute. *Biodiversity* is defined as the sum of structural and functional variation of 3 constituents—ecosystems, species, and genes (Pimm, 2021). Each component has numerous proxies (e.g., richness, evenness, rarity, heterozygosity) (Sarkar, 2016). The emergence of the term in the 1980s (Sarkar, 2021) provided a sense of a solution to the vagueness associated with nature and naturalness. It rapidly became a foundational concept in conservation science, creating an impression that the term is conceptually clear, empirically useful, and intrinsically valuable, and became the focus of conservation and a buzzword for better advocacy (Noss, 1990; Sarkar, 2002). *Biodiversity* is considered a better substitute for *nature* because it focuses directly on the impact of the crisis of the Anthropocene—namely, the loss of biodiversity—and should therefore be more effective in influencing decision makers and impressing the public (Oksanen, 2004; Takacs, 1996).

Nevertheless, *biodiversity* too has attracted substantial criticism regarding its meaning, value, and difficulties in articulating specific goals (Elliot, 2020; Faith, 2021a). First, as a goal of conservation, *biodiversity*, per se, is criticized as too complex or fuzzy. Meinard et al. (2019) attribute this to the definition of *biodiversity* that does not enable singling out a uniquely relevant sense (i.e., Which biodiversity? The richest? The most productive?). Although the vagueness of the biodiversity concept has been widely contemplated in philosophical literature, an unambiguous definition may not be attainable (Newman et al., 2017). As a metric, the fuzzy definition of biodiversity potentially results in 3 category errors (Maier, 2012, pp. 74–76): conflating condition with measure (e.g., species diversity = species richness), ignoring kinds (e.g., the species themselves), and generating the perception that diversity itself has value (i.e., more diverse means better, making non‐native invasions beneficial). Consequently, some objectives encompassed in the concept of nature conservation may be lost when replacing it with biodiversity conservation because the metrics appear unrelated to biodiversity. For example, a change in biomass in an ecosystem may, in theory, occur with no changes in species or genetic composition, yet the change affects ecosystem diversity. Second, as a buzzword, biodiversity is argued to have failed in galvanizing public attention and social or policy thinking among scientists (Devictor & Meinard, 2020; Elliot, 2019; Taylor, LeVasseur, et al., 2020). Third, controversy also surrounds the type of value under which biodiversity should be classified (Koricheva & Siipi, 2004; Sarkar, 2016): intrinsic, only instrumental, or only scientific value. Finally, in the extreme, biodiversity is claimed to be valueless (Santana, 2014, 2018), a poor metric of ecosystem attributes (Santana, 2016), and a failed political tool (Takacs, 1996, p. 99).

The many attitudes toward biodiversity combined with the sidelining of *nature* and *natural* have resulted in advocating a pluralistic approach to its possible values (Pascual et al., 2021). Within the ecocentric–intrinsic school, the belief that biodiversity per se has intrinsic value (reviewed by Elliott [2020]) dates back to 1985 (Soule, 1985) and is held by the Society for Conservation Biology (see the Society's Organizational Values at https://conbio.org/about‐scb/who‐we‐are#values). Elliott (2020) points out that the definition in the Convention on Biological Diversity does not address biodiversity's connection to humans—biodiversity is therefore apparently of little instrumental value and must therefore have intrinsic value. Yet, it is difficult to pinpoint what specifically about biodiversity holds this value (Linquist et al., 2020; Maier, 2012; McShane, 2016; Newman et al., 2017). Is the value in the biological (*bio*‐), in which case biocentrism provides a simpler alternative (Baard, 2021). Or, is the value in diversity (harmony through variety), which suggests the unacceptable conclusion that diversity should be artificially increased by introductions of non‐native species or genetically modified organisms (Heyd, 2010; Maier, 2012; Morar et al., 2015). Other arguments for intrinsic value rely on claims of ‘otherness’ (Wienhues & Deplazes‐Zemp, 2022) or irreplaceable design (Cline, 2020). Sarkar (2019) makes an analogy between the intrinsic value of biodiversity in conservation and the intrinsic value of human health in medicine. This analogy is confused, however, as we elaborate below.

The ecocentric school may also value biodiversity for its functional role as the driver of evolution and a contributor to the stability and resilience of ecosystems (Dereniowska & Meinard, 2021; Devictor & Meinard, 2020). Studies focusing on the biodiversity–stability relationship have produced equivocal findings, however (Wright et al., 2015), and the relationship, if it exists, may be bidirectional and nonmonotonic (Worm & Duffy, 2003). Thus, the functional value of biodiversity as a standalone concept cannot be generalized (e.g., the more biodiversity, the better). Another argument for biodiversity refers to its option value (Faith, 2021c), that is, its potential contribution to yet undiscovered medical and other valuable assets (Butkus, 2015). However, with recent advances in biosynthetic engineering, this argument is becoming moot (Linquist et al., 2020). A different argument is that biodiversity is a dynamic social construction, valued as a focal point for socioenvironmental causes (Haila, 2004). Still, the strength of biodiversity's social power is founded on its biological role (Görg, 2004), which remains contested.

In sum, use of *biodiversity* has not proven a remedy to the vagueness associated with the concept of nature and has not mitigated disagreements. The perplexity at the center of conservation philosophy therefore persists.

## INCORPORATING *NATURE*, *NATURAL*, AND *BIODIVERSITY* INTO A SINGLE CONSERVATION FRAMEWORK

### *Nature* and *natural* again

We offer a general scheme for the relation between *biodiversity* and *the natural* in conservation science. This scheme relies on the conservation ethic developed recently in Saltz and Cohen (2023), which recognizes naturalness as the ultimate value within the ecocentric–intrinsic school. First, we consider briefly how, in our view, the seeming conundrum regarding the meanings of *nature* and *natural* in conservation should be settled.

Nature conservation typically refers to the conservation of natural nature, not conservation of human artifacts. The aforementioned 2 definitions of *nature* that are relevant in conservation science—one referring to the totality of empirical reality and the other referring to the whole of material reality that is independent of human activity—can generate confusion. If one accepts the latter definition (nature as excluding art), then *natural nature* in which conservation science is interested becomes redundant and utterly uninformative. If, however, one accepts the former definition (*nature* as totality), then one is left with the nature–art versus natural–artificial conundrum. The ease with which one can slide to such confusions seems to have played a role in the motivation to abandon *nature* and *natural* in favor of *biodiversity*.

Semantic issues should not influence conservation philosophy, however. What one should rather say is that there are 2 possible nomenclatures, and the aim of conservation can be described by each. Either one views *nature* as the totality of empirical reality and concludes that there are 2 parts to nature, natural nature and artificial nature (as Mill [1874] concluded) (the former being separate from anthropogenic influence and the latter being anthropogenically influenced [Lanzerath, 2014]), or one understands nature as excluding human products, in which case, everything pertaining to nature is the business of conservation. Defining nature as the totality of empirical reality has been argued to recognize humans and their artifacts as part of the evolutionary process, which is a fundamental concept in conservation, and agrees with Leopold's view of humans as members of the ecological community (Callicott, 1987). Ultimately, however, which definition of *nature* one chooses is inconsequential to conservation. What is crucial is the distinction between the artificial and the natural (Elliot, 1992; Siipi, 2004). The latter—naturalness—is the subject matter of conservation, though its importance varies depending on the school of thought (ecocentric or anthropocentric).

### A 3‐tiered pluralist framework for conservation

Saltz and Cohen (2023) present a 3‐tier pluralist scheme for ethical thinking in conservation within the ecocentric–intrinsic school. At the top level is naturalness as the ultimate value. The middle level is composed of 3 principles: autonomy (freedom from anthropogenic interference), integrity (structural and functional completeness), and resilience (capacity to absorb external [anthropogenic] disturbances or changes without undergoing dramatic state shifts) of ecosystems. All these principles are reasonable interpretations of the duty to protect the overarching value of naturalness, allowing evolutionary processes to proceed as close as possible to their undisturbed trajectory (Robert et al., 2017). Hence, conservation thinking should be pluralist in the sense of not having to decide on one true principle for preserving naturalness over others. At the lower level are specific judgments that are sensitive to case‐specific conditions.

Given the plurality of midlevel principles, how are conservation decisions to be made? Necessarily, the principles are not absolute, but defeasible, such that to the extent that the application of different principles simultaneously is impossible, and one confronts a dilemma, one principle will yield to another or each will be only partly realized. The input from the lower level helps govern the prioritization of conservation principles in each specific case with the aim of maximizing naturalness. Instructively, this view of principle pluralism in conservation resembles the dominant approach in medical ethics, known as principlism (Beauchamp & Childress, 2019). Saltz and Cohen (2023) emphasize that decisions are the result of a holistic examination of the specific considerations that pertain to each (type of) case. Ethical judgment regarding the balancing of principles against one another is a function of ethical sensitivity to specific considerations and of practical wisdom—an ethical know‐how that resists formalization. This bottom‐up element complements the typical top‐down orientation of ethical judgment (i.e., application of principles to specific judgments). This bidirectional scheme allows for optimal coherence in decision‐making. Furthermore, the fact that each of the conservation principles is a valid embodiment of the one supreme value of naturalness allows for unity within a pluralist scheme of decision‐making.

### Naturalness and biodiversity in the context of principle pluralism

Given that naturalness is the ultimate value of the ecocentric–intrinsic school, what is the role of biodiversity in this scheme? Saltz and Cohen (2023) do not explain. This is a glaring omission in the 3‐tiered pluralist conservation scheme. Here, we add this crucial component in a way that fits snugly with the existing components and makes the entire theory complete.

The ultimate moral value of naturalness explicates the desired state of an area (possibly the entire globe). The current state of the land and how much it deviates from the desired state provide the evidence necessary for decision‐making and constructing management protocols. Within this framework, biodiversity is an umbrella concept for the metrics necessary to assess the naturalness of an area, gauge deviations from this goal, and assess proximity to state‐shift thresholds (Santana, 2016). In Saltz and Cohen (2023), adjudication between the midlevel principles of conservation does not have a formal structure and is left to intuition. However, these principles refer directly to biodiversity, its constituents, and the factors affecting them (connectivity, size, and shape). Thus, biodiversity ought to be added as a structural component to the decision‐making scheme, providing the basis for decisions regarding which midlevel principle to prioritize.

This fits well with the main example discussed by Saltz and Cohen (2023): the possible reintroduction of a locally extirpated species—a conservation procedure that is, relative to other conservation protocols, an extreme violation of the ecosystem's and animals’ autonomy. Some of the main questions that conservationists ought to contemplate follow. What is the world status of the species (size and number of other wild populations)? Is reestablishment by natural dispersal from other populations feasible? Is the species keystone and will its absence cause loss of structural and functional biodiversity due to cascading effects? What is the probability of irreversible cascading impacts before reestablishment by natural dispersal? Will the species’ extirpation make the ecosystem susceptible to invasion by non‐native species or reduce its resilience to future global changes? How much anthropogenic involvement is necessary for a successful reintroduction (e.g., provision of supplemental resources that may impact other species)? The crucial point is that all these questions refer directly to the constituents and proxies of biodiversity or require their considerations. An example is the contribution of reintroduced ungulates to long‐distance dispersal and germination of seeds (Polak et al., 2014). Although these reintroductions clearly violate the autonomy of the system and can be viewed as an artifact, they contribute to the natural biodiversity (integrity) of the ecosystem beyond the restoration of the species being reintroduced and thus outweigh the violation of autonomy. The framework suggested here acknowledges the crucial role of biodiversity as a component in the protocol of protecting and restoring naturalness (Faith, 2021b). It does not, however, accord biodiversity the status of ultimate value.

If biodiversity measurements direct the choice of midlevel principles, is not the employment of “moral sensitivity” and “practical wisdom” (Saltz & Cohen, 2023) made redundant? It is not. This is because, as a metric, biodiversity is multidimensional, with 3 constituents each requiring proxies, some of which are not quantifiable (Maclaurin & Sterelny, 2008). The proxies that are quantifiable are incommensurable—they cannot be projected onto a single scale (Habib, 2015; Koricheva & Siipi, 2004; Purvis & Hector, 2000). As Bryan Norton (2008, p. 17) sums this up: “Biodiversity cannot be defined in such a way as to make it a measurable quantity. That is, we cannot provide an index allowing us to rate ecosystems or collections of entities according to their degree of diversity.” Because biodiversity cannot be reduced to one commensurable statistic, the necessity of practical wisdom of conservationists, based on sensitive ethical judgment of case specifics (e.g., prioritizing the various proxies of biodiversity and response options available), remains crucial to the decision‐making scheme (see related insight in Haila [2004, p. 64]).

Even if hypothetically, one could reach an objective all‐things‐considered biodiversity score for an ecosystem, one would still face the following problem. To the extent that autonomy (a conservation principle) has independent value, then, even if active management to maintain integrity (a different conservation principle) would yield a higher overall biodiversity score than self‐recovery, one would still need to make a value judgment regarding the size of the gap between the 2 conservation principles that justifies intervention. Thus, for example, if integrity gets a 75 overall biodiversity score and autonomy gets 73, one would need to judge whether a gap of merely 2 points is sufficient to justify active intervention, or whether one should nonetheless prioritize the midlevel principle of autonomy. Again, ethical judgment grounded in practical wisdom would remain indispensable, even under scientifically utopian conditions.

In a conservation philosophy that does not recognize principle pluralism, biodiversity as a salient parameter, trivially, could not have the function of adjudicating between conservation principles. The role of biodiversity in such a philosophy would remain vague or unclear. If that philosophy adopted only the principle of autonomy, biodiversity might have no systematic role to play. If it adopted only the principle of integrity or of resilience, it would be easy to erroneously conflate biodiversity and naturalness.

The role of biodiversity in conservation science can be further elucidated through an analogy to medicine (a rather common analogy [Ehrenfeld, 2000]). The analogy is best appreciated by inspecting the relations among factors in the general scheme of: agent, object of care, ultimate value to be realized, and salient parameter examined. In medicine, this is manifested as: medical professional ↔ patient (individual) ↔ health (well‐being) ↔ maintaining or achieving homeostatic functions. For the ecocentric–intrinsic value school of conservation, with which we are dealing, the analogy is: conservation professional ↔ nature (specific area) ↔ naturalness ↔ maintaining or achieving natural biodiversity. Here, naturalness is analogous to health, and, like health, it is not fully attainable. Our analogy is importantly different from previous understandings. Odenbaugh (2021) writes: “many have likened [conservation biology] to medicine insofar as it has ethical foundations. In the medical case, it would be the well‐being of patients and in conservation biology it is the conservation of biodiversity.” Biodiversity is here analogized to health (see also Sarkar [2002]), thus exhibiting the status of ultimate value. In our scheme, in contrast, the ultimate value of naturalness (in conservation) is analogous to the ultimate value of health (in medicine), and biodiversity is the salient parameter with which to evaluate the realization of the value, analogous to homeostasis in medicine.

Clearly, health, not homeostasis, is the ultimate value in medicine. Homeostasis is the most salient empirical test aggregating the parameters that monitor physical health and recovery from ailments. Accordingly, doctors may accept changes of homeostasis if they improve health. For example, if seeking to lower the blood pressure of a hypertensive patient to normal levels would risk bouts of dangerous hypotension, then the doctor might settle on a somewhat higher average blood pressure as a new homeostatic optimum (this has a parallel logic to the principle of resilience in conservation). Complementarily, there can be a disruption of health even without measured alteration of homeostasis (e.g., phantom limb pain, which a doctor would treat). These insights reinforce our claim that the function of biodiversity in conservation is parallel to that of homeostasis in medicine, not (as has been claimed) of health.

Patients, like specific land sections, can be viewed evolutionarily, where different organs have been selected to function best within specific boundaries of various physiological parameters (homeostasis), such as blood pressure, hematocrit, or body temperature (Ananth, 2008). Natural ecosystems exist in a dynamic equilibrium, and under a natural disturbance regime, their state conceptually resembles a homeostatic state where the proxies of biodiversity are expected to fall within certain boundaries. Anthropogenically induced deviations from these boundaries are indicative of reduced naturalness and can lead to state shifts if certain thresholds are crossed (Huggett, 2005). In contrast to homeostasis that is actively maintained by physiological processes, the dynamic equilibrium of a natural ecosystem is maintained by negative feedback between various biodiversity components. This phenomenon bares similarity to a homeostatic property cluster (HPC) (Burch‐Brown & Archer, 2017), which is maintained when mechanisms ensure that components have reduced persistence outside the cluster. Biodiversity per se is, therefore, not an HPC (Santana, 2018). However, the natural biodiversity of a given area at a time scale where fluctuations of environmental conditions are consistent does maintain a dynamic equilibrium and can be considered akin to an HPC. Thus, when referring to natural biodiversity or its components (e.g., natural habitat being analogous to healthy blood parameters), one is essentially referring to HPC‐like conditions where numerous measurable attributes (e.g., species richness, abundance, heterozygosity in an area designated for conservation, or erythrocyte and leucocyte counts in a patient's blood) are expected to be maintained within specific boundaries for the ecosystem to autonomously maintain its dynamic equilibrium.

In sum, biodiversity shows deep parallelism with homeostasis; just as homeostasis is the central marker of health, biodiversity is the central marker of naturalness. Viewing biodiversity in this light is arguably rather intuitive. The term *biodiversity* on its own can be interpreted as a normative goal or as an umbrella concept for the metrics necessary to assess the current versus the desired (natural) state of an area. The two are often conflated (one of Maier's [2012] 3 category errors). If biodiversity is a goal, then the ultimate value would be its maximization, making the construction of novel ecosystems (enhanced ecosystem diversity) flush with non‐native species (enhanced species diversity) and genetically modified organisms (enhanced genetic diversity) a desired outcome (e.g., Lean, 2021; Mychajliw et al., 2022). This clearly is not the desired conservation goal (Angermeier, 1994). Accordingly, biodiversity as a standalone holds little ethical value, and attempts to view biodiversity as the apex of conservation efforts face justified scrutiny (Maier, 2012; Santana, 2018; Takacs, 1996). If biodiversity is an umbrella concept for conservation metrics in a specific area, then the desired biodiversity state must be singled out by a qualifier reflecting a unique ultimate value of interest (Meinard et al., 2019; Santana, 2019). Specifically, the qualifier should describe a unique corporate of organisms and environment. For the intrinsic value school, the obvious qualifier is natural (i.e., natural biodiversity) (Saltz & Cohen, 2023), which specifies a unique condition for a given area. Furthermore, it embodies a definitive norm. Indeed, conservation efforts may be geared toward decreasing unnatural biodiversity. *Natural* as a qualifier of biodiversity indeed appears in the first organizational value of the Society for Conservation Biology (“*There is intrinsic value in the natural diversity of organisms…*” [SCB, 2011]) and is a common qualifier of biodiversity‐related terms (e.g., *natural habitat*) in conservation studies (Saltz & Cohen, 2023). Naturalness confers intrinsic value to biodiversity (Elliott, 2020). Regretfully, often the *natural* qualifier is only implied. For example, when Sarkar (2002) states, “the task of conservation is to conserve biodiversity,” he is arguably referring to natural biodiversity. Omitting the qualifier leaves the type of biodiversity open to Maier's (2012) category errors.

## CONCLUSIONS

Although replacing the term *nature conservation* with *biodiversity conservation* afforded a sense of explicitness, it also resulted in the false recognition of biodiversity in and of itself as an ultimate value of the ecocentric school. An encyclopedia entry on conservation biology asserts: “conservation biology as a discipline has expended a great deal of intellectual effort in articulating exactly what is its object of study and has settled on biodiversity as the answer” (Odenbaugh, 2021). Whether this settling on biodiversity is appropriate depends on how one interprets *object of study*. If one understands it as referring to the central parameter to be measured, then it is wholly appropriate. If one understands it as specifying the dominant value of this normative science, it is inappropriate. In fact, the value goals of ecocentric conservation can be stated, defined, and expounded independent of the term *biodiversity*. For example, in a review of biodiversity as a concept (Díaz & Malhi, 2022), in a section titled “The Diverse Values of Biodiversity,” *biodiversity* is used only once, whereas *nature* appears 14 times, all in the context of values. In a paper titled “What Is Conservation?”, Sandbrook (2015) makes no mention of biodiversity. In a paper focusing on the ontology of the 3 ultimate values of conservation in the context of ethical pluralism (Pascual et al., 2017), *biodiversity* appears only 3 times, and none of the uses refers to value.

The polysemy of *nature* and *natural* should not hinder progress in conservation science. Naturalness should be defined as that part of nature where biodiversity remains free from human impact and recognized as the ultimate moral value within the ecocentric–intrinsic school. *Biodiversity* is an umbrella term for the metrics necessary for quantitatively and qualitatively defining and assessing success in achieving this ultimate value. As such, biodiversity is a vital concept in conservation science because, like homeostasis, it enables conservation scientists to focus research on key issues and assist decision makers in prioritizing between midlevel principles. Nevertheless, it is important to realize that biodiversity fails to hold the attributes of a fundamental norm and cannot inform decision‐making unless the type of biodiversity sought is specified (e.g., natural biodiversity). Conserving naturalness and conserving biodiversity should not represent different schools of thinking—the former obsolete, the latter new and improved. Rather, they are parts of the same ethical framework. Naturalness is the ultimate goal, and biodiversity is the proximate goal that encompasses the metrics used to assess and approach naturalness.
